# Investigating the Role of Gut Microbiota in the Pathogenesis and Progression of Rheumatoid Arthritis in a Collagen-Induced Arthritis Mouse Model

**DOI:** 10.3390/ijms26115099

**Published:** 2025-05-26

**Authors:** Paulína Belvončíková, Kristína Macáková, Nikola Tóthová, Pavel Babál, Lenka Tarabčáková, Roman Gardlík

**Affiliations:** 1 Institute of Molecular Biomedicine, Faculty of Medicine, Comenius University, Sasinkova 4, 811 08 Bratislava, Slovakia; belvoncikova2@uniba.sk (P.B.); macakova28@uniba.sk (K.M.); tothova340@uniba.sk (N.T.); 2 Institute of Pathological Anatomy, Faculty of Medicine, Comenius University, Sasinkova 4, 811 08 Bratislava, Slovakia; pavel.babal@fmed.uniba.sk; 3 Department of Rheumatology, Saint Michael’s Hospital, Satinského 1, 811 08 Bratislava, Slovakia; lenka.tarabcakova@gmail.com

**Keywords:** gut microbiota, fecal microbiota transplantation, autoimmunity, inflammation, rheumatoid arthritis

## Abstract

Rheumatoid arthritis (RA) is a chronic systemic autoimmune disorder whose precise etiology remains unclear, though growing evidence implicates gut microbiota in its pathogenesis. This study aimed to investigate the role of gut microbiota in the onset and progression of RA by employing fecal microbiota transplantation (FMT) in a collagen-induced arthritis (CIA) mouse model using DBA/1J and Aire^−^/^−^ strains. Mice received FMT from healthy donors, treatment-naïve RA patients, or treated RA patients in relapse, followed by assessment of microbiota composition via 16S rRNA sequencing, arthritis severity scoring, histological evaluations, and systemic inflammatory markers. The findings revealed distinct microbiota clustering patterns post-FMT across experimental groups, highlighting strain-specific colonization effects. Notably, genera such as Bifidobacterium and Paraprevotella correlated positively with arthritis severity in DBA/1J mice, whereas Corynebacterium, Enterorhabdus, and Odoribacter exhibited negative correlations, suggesting potential protective roles. Despite these microbial differences, minor variations in arthritis scores, paw inflammation, or systemic inflammation were observed among FMT groups. This indicates that although gut microbiota alterations are associated with RA pathogenesis, further investigation with larger cohorts and comprehensive sequencing approaches is essential to elucidate the therapeutic potential of microbiome modulation in autoimmune diseases.

## 1. Introduction

Rheumatoid arthritis (RA) is a chronic systemic autoimmune disease that typically manifests as progressive joint inflammation. In seropositive patients, its pathogenesis is characterized by the presence of anti-citrullinated protein antibodies (ACPAs) and rheumatoid factor (RF), both of which play a pivotal role in disease progression [1]. In addition to autoimmune antibodies, another hallmark of RA is elevated levels of proinflammatory cytokines [2]. Although the pathogenesis of RA has been described, its exact etiology remains unclear [1]. Key risk factors include genetic predisposition, sex, and environmental and lifestyle influences, such as smoking [3].

An increasing number of studies have highlighted the role of human microbiota in RA pathogenesis, particularly the impact of oral microbiota alterations and periodontal diseases [4,5,6]. *Porphyromonas gingivalis*, a key periodontopathogen, appears to be implicated in RA pathogenesis by triggering autoimmune responses through protein citrullination. This process can lead to the mistaken recognition of these proteins as foreign by the immune system, resulting in the production of ACPAs. These antibodies subsequently contribute to chronic inflammation and joint damage [7].

However, immune system stimulation can originate from microbiota inhabiting not only the oral cavity but also other body sites. Fewer studies focus on the role of gut microbiota in the pathogenesis of RA, despite the fact that it represents the most densely populated and complex ecosystem within the human body [8]. The mechanism by which gut microbiota dysbiosis may promote systemic inflammation in RA involves disrupting the gut–joint axis [9]. Gut bacteria produce metabolites, such as short-chain fatty acids, which can modulate immune system responses—leading to increased production of proinflammatory cytokines and altering the balance of immune responses, similar to what occurs in RA. Gut dysbiosis and the subsequent microbial products of pathogenic bacteria may spread into circulation, reach distant organs, and promote low-grade systemic inflammation [10,11,12]. This dysbiosis-induced disruption of immune regulation has been linked to the onset and worsening of autoimmune conditions, including RA [13,14].

Changes in gut microbiota composition have been reported in numerous studies [15,16]. Specific microbial genera, such as *Prevotella*, have been linked to an increased risk and severity of RA through modulation of the immune response and inflammatory pathways [17]. Conversely, bacteria such as *Akkermansia* and *Bifidobacterium* have been shown to have protective effects by promoting anti-inflammatory responses, restoring gut homeostasis, and potentially mitigating disease progression [18]. Therefore, the gut microbiota represents an important factor that may help to explain the complex interplay between environmental triggers and the pathogenesis of RA.

These findings have spurred interest in therapeutic strategies targeting the gut microbiota, including fecal microbiota transplantation (FMT). FMT involves the transfer of gut bacteria from a healthy donor into a recipient’s gut and has emerged as a promising approach to restoring microbial homeostasis [19]. Initially applied for the treatment of recurrent *Clostridioides difficile* infections, FMT has shown potential positive effects in clinical trials for autoimmune diseases, including psoriatic arthritis and celiac disease [20,21,22]. In the context of RA, early studies suggest that FMT may also help alleviate disease symptoms [23].

Animal models of RA are essential tools for understanding the disease’s pathogenesis and testing potential therapeutic interventions. These models mimic various aspects of RA, including immune system dysregulation, joint inflammation, and tissue destruction. One of the most widely used models is the collagen-induced arthritis (CIA) model, which is developed by immunizing susceptible mouse or rat strains with type II collagen emulsified in Freund’s adjuvant, inducing an autoimmune response resembling human RA [24]. The most commonly used strain in the CIA model is DBA/1J mice [25,26,27]. However, the use of mouse strains with different immune system dysregulations could help elucidate the role of gut microbiota in specific autoimmune pathways. The Aire^−^/^−^ mouse strain represents a novel strategy for studying the pathogenesis of RA. The transcription factor AIRE plays a key role in autoimmunity by promoting the ectopic expression of peripheral tissue-restricted antigens in the medullary epithelial cells of the thymus. In Aire^−^/^−^ mice, a transcript may be generated from the targeted locus, but a functional protein is not translated, leading to lymphocytic infiltrates in organs and the presence of serum autoantibodies. As a result, Aire^−^/^−^ mice develop broad defects in immunological tolerance and a variety of autoimmune diseases [28].

The primary aim of this study is to explore the role of gut microbiota in the onset and progression of RA using the CIA model combined with the FMT method. Insights gained from this study may pave the way for microbiome-targeted therapies, offering novel treatment strategies to improve patient outcomes in autoimmune diseases.

## 2. Results

### 2.1. Gut Microbiota Differences

**Figure 1:**
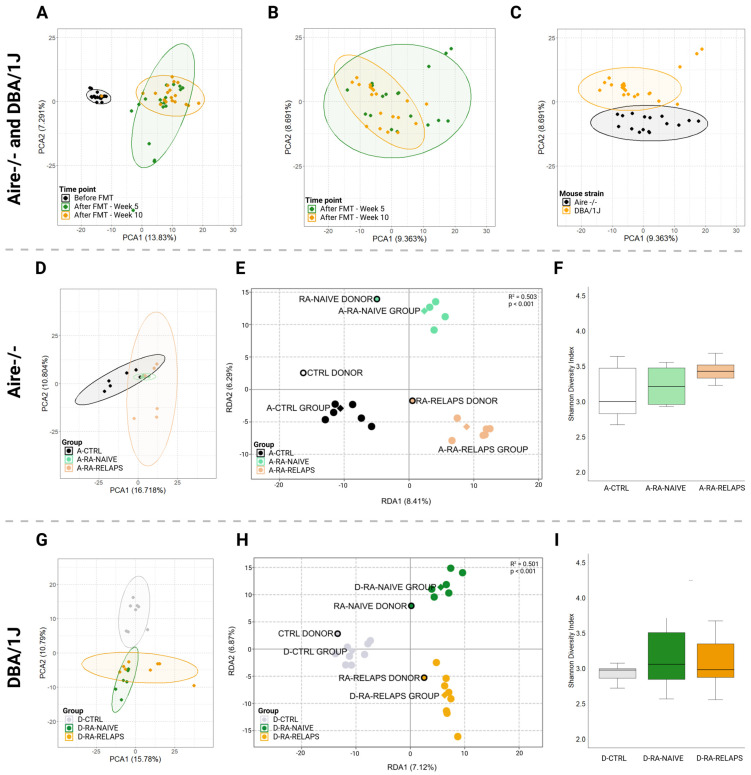
Gut microbiota clustering and alpha diversity index: (**A**) Principal component analysis (PCA) plot of all mouse recipient samples across various time points, (**B**) PCA plot of all mouse recipient samples across post-fecal microbiota transplantation (FMT) time points, (**C**) PCA plot of all mouse recipient samples across mouse strains, (**D**) PCA plot of Aire^−^/^−^ mouse recipient samples across various FMT received, (**E**) Redundancy analysis (RDA) plot of donors pools and Aire^−^/^−^ mouse recipient samples across various FMT received, (**F**) Shannon diversity index of Aire^−^/^−^ mouse recipient samples across various FMT received, (**G**) PCA plot of DBA/1J mouse recipient samples across various FMT received, (**H**) RDA plot of donor pools and DBA/1J mouse recipient samples across various FMT received, (**I**) Shannon diversity index of DBA/1J mouse recipient samples across various FMT received. All data presented are analyzed at the amplicon sequence variant level. Boxplot data are presented as median, interquartile range, and minimum and maximum (**F**,**I**); solid dot—mouse recipient samples; framed dot—donor pool samples; diamond—group average; ellipse—confidence interval; PCA1—first principal component with the most variance; PCA2—second principal component with the second most variance; RDA1—first explanatory variable with the most variance; RDA2—second explanatory variable with the second most variance; A-CTRL—Aire^−^/^−^ mice that received FMT from healthy donors; A-RA-NAIVE—Aire^−^/^−^ mice that received FMT from naive rheumatoid arthritis (RA) patients, A-RA-RELAPS—Aire^−^/^−^ mice that received FMT from treated RA patients in relapse; D-CTRL—DBA/1J mice that received FMT from healthy donors; D-RA-NAIVE—DBA/1J mice that received FMT from naive RA patients, D-RA-RELAPS—DBA/1J mice that received FMT from treated RA patients in relapse.

The sequencing produced an average of 83,173 ± 9469 raw reads per sample, with the lowest read count being 68,466. After quality filtering and chimera removal, an average of 47,397 ± 5190 high-quality reads per sample remained, with a minimum of 38,824 reads. From these, ASV were generated, and taxonomic classification was performed at the genus level. Unassigned reads at the genus level, representing 6.25 ± 1.45% of the total reads, were removed prior to downstream microbiome analysis. To examine the role of gut microbiota in the pathogenesis of RA, 16S rRNA gene amplicon sequencing was performed. Principal component analysis (PCA) of recipient samples showed distinct clustering patterns on the amplicon sequence variant (ASV) level corresponding to time points pre- and post-FMT application (Figure 1A). Interestingly, post-FMT samples, week 5 and week 10, exhibited similar microbial composition, as seen by clusters overlap (Figure 1A,B). Further analysis of post-FMT time points revealed strain-specific microbiome clustering (Figure 1C). When analyzing individual mouse strains, PCA analysis of the Aire^−^/^−^ recipient mice showed clustering of the samples corresponding to the specific FMT received, specifically transplants from healthy controls and treated RA patients in relapse (Figure 1D). In contrast, mice receiving FMT transplants from naive RA patients overlapped with other groups (Figure 1D). A redundancy analysis (RDA), including all donor pools and post-FMT recipient samples, was performed to verify a successful FMT procedure. This revealed a distinct transplantation-specific clustering across donors and Aire^−^/^−^ mice (R^2^ = 0.503, *p* < 0.001, 1000 permutation test; Figure 1E). Alpha diversity analysis, represented by the Shannon index, showed no differences in microbial richness and evenness of the Aire^−^/^−^ mice post-FMT (*p* = ns; Figure 1F). Correspondingly, the PCA analysis of the DBA/1J mice showed specific clustering patterns across different FMTs transplanted (Figure 1G). Mice that received FMT transplants from healthy donors clustered separately, while samples from post-FMT mice that received transplants from patients overlapped. Importantly, donor pools and DBA/1J mouse recipients post-FMT clustered alike in RDA analysis, suggesting successful FMT procedures (R^2^ = 0.501, *p* < 0.001, 1000 permutation test; Figure 1H). In DBA/1J mice, the Shannon index did not differ across the groups post-FMT (*p* = ns; Figure 1I).

**Figure 2:**
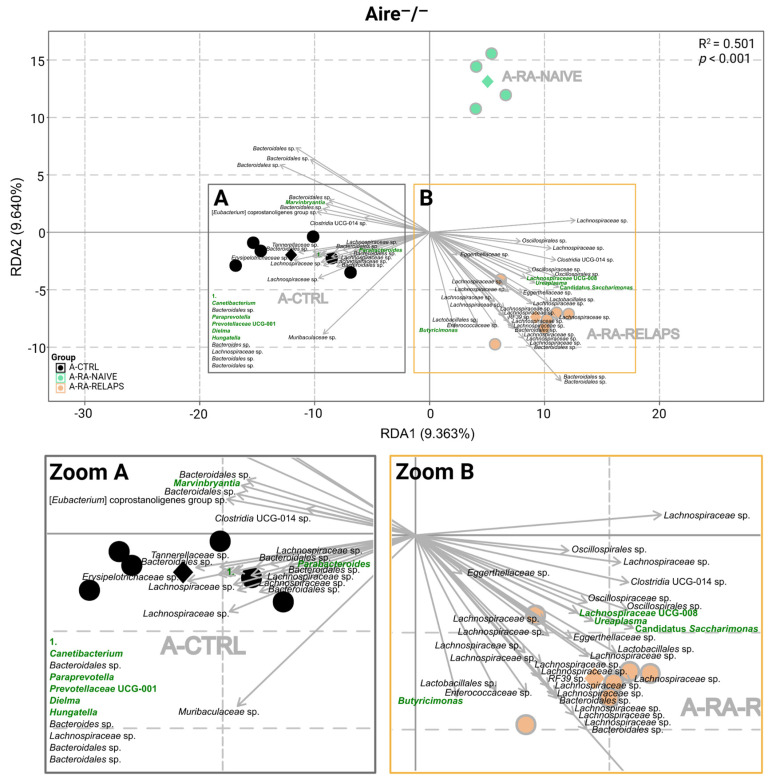
Redundancy analysis (RDA) of gut microbiota in Aire^−^/^−^ mouse recipients: RDA analysis plot of Aire^−^/^−^ mouse recipient samples across various fecal microbiota transplants (FMT) received, (**A**) Zoom of the A square, (**B**) Zoom of the B square. All data presented are analyzed at the genus level with *p* < 0.01; green highlighted text—significant FMT-specific genera; black text—other significant FMT-specific genera with no identified genus taxonomy; grey text—group description; black dot—A-CTRL mouse recipient samples; black diamond—A-CTRL group average; green dot—A-RA-NAIVE mouse recipient samples; green diamond—A-RA-NAIVE group average; orange dot—A-RA-RELAPS mouse recipient samples; orange diamond—A-RA-RELAPS group average; arrow—distribution of individual microbial taxa in which the direction and length reflect the group and strength of association, respectively; RDA1—first explanatory variable with the most variance; RDA2—second explanatory variable with the second most variance; A-CTRL—Aire^−^/^−^ mice that received FMT from healthy donors; A-RA-NAIVE—Aire^−^/^−^ mice that received FMT from naive rheumatoid arthritis (RA) patients, A-RA-RELAPS—Aire^−^/^−^ mice that received FMT from treated RA patients in relapse.

RDA analysis was carried out to analyze gut microbiota composition at the genus level. In Aire^−^/^−^ mice, RDA analysis showed significant differences in microbial composition across groups (R^2^ = 0.501, *p* < 0.001, 1000 permutation test; Figure 2), specifically in genera *Butyricimonas*, *Marvinbryantia*, *Ureaplasma*, *Prevotellaceae* UCG-001, *Paraprevotella*, Candidatus *Saccharimonas*, *Lachnospiraceae* UCG-008, *Hungatella*, *Catenibacterium*, *Parabacteroides*, and *Dielma* (*p* < 0.01; Figure 2, Table S1A). Minor, but relevant, changes were also observed in genera *Bifidobacterium*, *Parasutterella*, *Lachnospiraceae* FCS020 group, *Enterorhabdus*, *Allobaculum*, *Monoglobus*, *Anaeroplasma*, *Tuzzerella*, *Coprobacter*, *Romboutsia*, *Turicibacter*, *Barnesiella*, *Enterobacter*, and [*Ruminococcus*] gauvreauii group (*p* < 0.05; Table S1A). No differences were seen across the genera of periodontopathogens *Prevotella* and *Prevotellaceae* NK3B31 group (*p* = ns; Table S1A).

**Figure 3:**
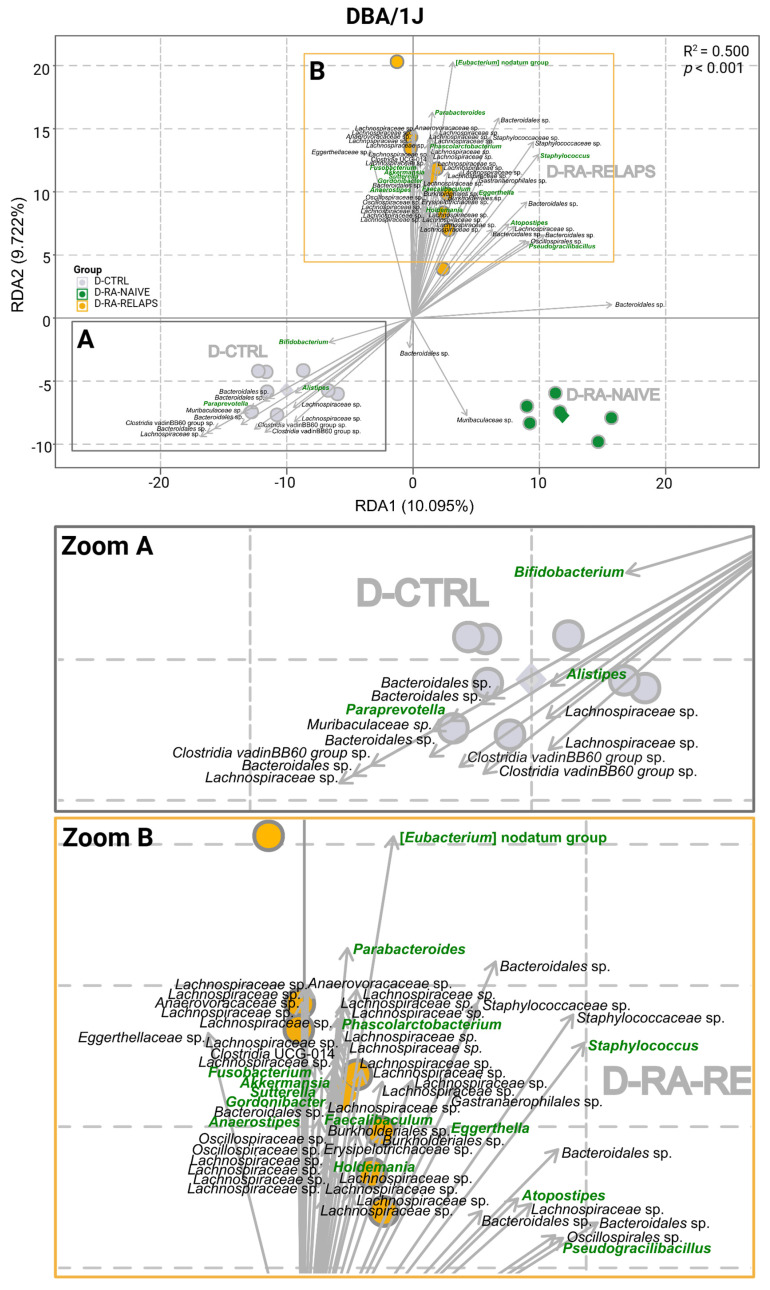
Redundancy analysis (RDA) of gut microbiota in DBA/1J mouse recipients: RDA plot of DBA/1J mouse recipient samples across various fecal microbiota transplants (FMT) received, (**A**) Zoom of the A square, (**B**) Zoom of the B square. All data presented are analyzed at the genus level with *p* < 0.01; green highlighted text—significant FMT-specific genera; black text—other significant FMT-specific genera with no identified genus taxonomy; grey text—group description; grey dot—D-CTRL mouse recipient samples; grey diamond—D-CTRL group average; green dot—D-RA-NAIVE mouse recipient samples; green diamond—D-RA-NAIVE group average; orange dot—D-RA-RELAPS mouse recipient samples; orange diamond—D-RA-RELAPS group average; arrow—distribution of individual microbial taxa in which the direction and length reflect the group and strength of association, respectively; RDA1—first explanatory variable with the most variance; RDA2—second explanatory variable with the second most variance; D-CTRL—DBA/1J mice that received FMT from healthy donors; D-RA-NAIVE—DBA/1J mice that received FMT from naive rheumatoid arthritis (RA) patients, D-RA-RELAPS—DBA/1J mice that received FMT from treated RA patients in relapse.

The RDA analysis was performed in DBA/1J mice at the genus level as well (R^2^ = 0.500, *p* < 0.001, 1000 permutation test; Figure 3). The most significant clustering pattern was seen in [*Eubacterium*] nodatum group, *Pseudogracilibacillus*, *Phascolarctobacterium*, *Bifidobacterium*, *Eggerthella*, *Paraprevotella*, *Akkermansia*, *Fusobacterium*, *Anaerostipes*, *Sutterella* (*p* < 0.001; Figure 3, Table S1B), but also *Alistipes*, *Parabacteroides*, *Staphylococcus*, *Gordonibacter*, *Atopostipes*, *Faecalibaculum*, and *Holdemania* (*p* < 0.01; Figure 3, Table S1B). Minor differences were observed in the genera *Corynebacterium*, *Facklamia*, *Marvinbryantia*, [*Eubacterium*] ventriosum group, *Odoribacter*, Candidatus *Stoquefichus*, *Parasutterella*, *Barnesiella*, *Allobaculum*, [*Clostridium*] innocuum group, and *Hungatella* (*p* < 0.05; Table S1B). No differences were seen across the genera of periodontopathogens *Prevotellaceae* UCG-001 and *Prevotellaceae* NK3B31 group (*p* = ns; Table S1B).

**Figure 4:**
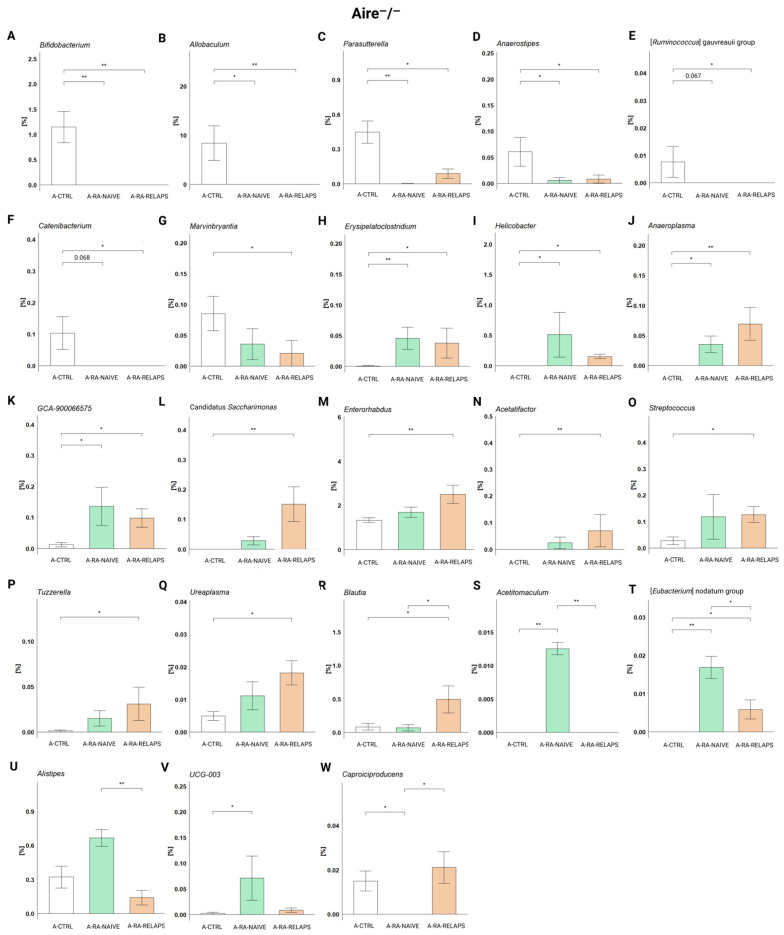
Relative abundances in Aire^−^/^−^ mouse recipients: (**A**) *Bifidobacterium*, (**B**) *Allobaculum*, (**C**) *Parasutterella*, (**D**) *Anaerostipes*, (**E**) [*Ruminococcus*] gauvreauii group, (**F**) *Catenibacterium*, (**G**) *Marvinbryantia*, (**H**) *Erysipelatoclostridium*, (**I**) *Helicobacter*, (**J**) *Anaeroplasma*, (**K**) *GCA-900066575*, (**L**) Candidatus *Saccharimonas*, (**M**) *Enterorhabdus*, (**N**) *Acetifactor*, (**O**) *Streptococcus*, (**P**) *Tuzzerella*, (**Q**) *Ureaplasma*, (**R**) *Blautia*, (**S**) *Acetitomaculum*, (**T**) [*Eubacterium*] nodatum group, (**U**) *Alistipes*, (**V**) *UCG-003*, and (**W**) *Caproiciproducens*. All data presented are analyzed at the genus level. Data are presented as mean ± SEM; * *p* < 0.05, ** *p* < 0.01; y axis—relative abundance in %; A-CTRL—Aire^−^/^−^ mice that received FMT from healthy donors; A-RA-NAIVE—Aire^−^/^−^ mice that received FMT from naive rheumatoid arthritis (RA) patients, A-RA-RELAPS—Aire^−^/^−^ mice that received FMT from treated RA patients in relapse.

Analyzing the relative abundances across groups in Aire^−^/^−^ strain, specific genera were predominantly present in the CTRL-transplanted mice. These were mainly *Bifidobacterium* (*p* < 0.001), but also *Allobaculum*, *Parasutterella*, *Anaerostipes* (*p* < 0.01), and [*Ruminococcus*] gauvreauii group, *Catenibacterium*, and *Marvinbryantia* (*p* < 0.05; Figure 4A–G, Table S1C). The post hoc Dunn’s test with Bonferroni correction showed that *Bifidobacterium*, *Allobaculum*, *Parasutterella*, and *Anaerostipes* were absent or significantly decreased in the A-RA-NAIVE as well as A-RA-RELAPS groups (*p* < 0.05; Figure 4A–D, Table S1C), while genera [*Ruminococcus*] gauvreauii group, *Catenibacterium*, and *Marvinbryantia* were only in the A-RA-RELAPS group, as compared to the group transplanted with FMT from healthy individuals (*p* < 0.05; Figure 4E–G, Table S1C).

In contrast, several genera were more prevalent in RA-pool transplanted mice. Specifically, these were *Erysipelatoclostridium*, *Helicobacter*, *Anaeroplasma*, Candidatus *Saccharimonas* (*p* < 0.01) and GCA-900066575, *Enterorhabdus*, *Acetifactor*, *Streptococcus*, *Tuzzerella*, *Ureaplasma*, and *Blautia* (*p* < 0.05; Figure 4H–R, Table S1C). While *Erysipelatoclostridium*, *Helicobacter*, *Anaeroplasma*, and GCA-900066575 were significantly more abundant in both groups that had received FMT from RA patients (*p* < 0.05; Figure 4H–K, Table S1C), genera Candidatus *Saccharimonas*, *Enterorhabdus*, *Acetatifactor*, *Streptococcus*, *Tuzzerella*, and *Ureaplasma* were more abundant only in the A-RA-RELAPS group, as compared to control FMT (*p* < 0.05; Figure 4L–R, Table S1C). Unlike other genera, *Blautia* was highly specific only for the A-RA-RELAPS group (*p* < 0.05; Figure 4R, Table S1C).

Several unique genera were identified in the A-RA-NAIVE group, such as *Acetitomaculum* (*p* < 0.001), [*Eubacterium*] nodatum group (*p* < 0.01), and *Alistipes,* UCG-003 (*p* < 0.05; Figure 4S–V, Table S1C). Notably, genus *Caproiciproducens* was completely absent in A-RA-NAIVE group (*p* < 0.05; Figure 4W, Table S1C).

**Figure 5:**
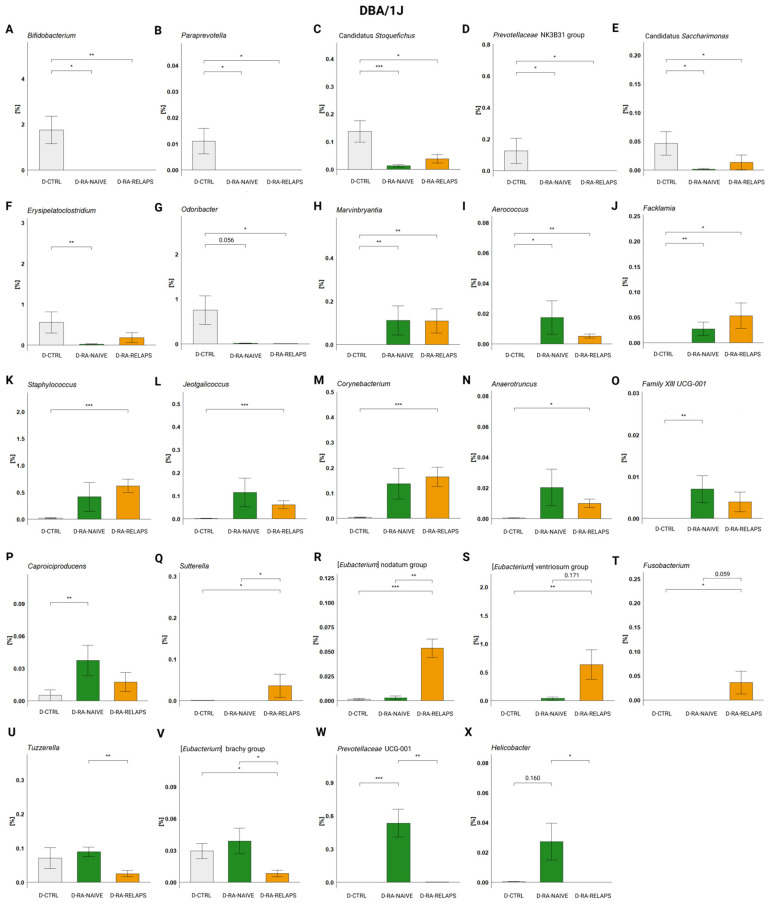
Relative abundances in DBA/1J mouse recipients: (**A**) *Bifidobacterium*, (**B**) *Paraprevotella*, (**C**) Candidatus *Stoquefichus*, (**D**) *Prevotellaceae* NK3B31 group, (**E**) Candidatus *Saccharimonas*, (**F**) *Erysipelatoclostridium*, (**G**) *Odoribacter*, (**H**) *Marvinbryantia*, (**I**) *Aerococcus*, (**J**) *Facklamia*, (**K**) *Staphylococcus*, (**L**) *Jeotgalicoccus*, (**M**) *Corynebacterium*, (**N**) *Anaerotruncus*, (**O**) *Family XII UCG-001*, (**P**) *Caproiciproducens*, (**Q**) *Sutterella*, (**R**) [*Eubacterium*] nodatum group, (**S**) [*Eubacterium*] ventriosum group, (**T**) *Fusobacterium*, (**U**) *Tuzzerella*, (**V**) [*Eubacterium*] brachy group, (**W**) *Prevotellaceae* UCG-001, and (**X**) *Helicobacter*. All data presented are analyzed at the genus level. Data are presented as mean ± SEM; * *p* < 0.05, ** *p* < 0.01, *** *p* < 0.001; y axis—relative abundance in %; D-CTRL—DBA/1J mice that received FMT from healthy donors; D-RA-NAIVE—DBA/1J mice that received FMT from naive rheumatoid arthritis (RA) patients, D-RA-RELAPS—DBA/1J mice that received FMT from treated RA patients in relapse.

In DBA/1J mice, specific genera were present preferably in the CTRL-transplanted mice too. Correspondingly to the RDA analysis, these were genera *Bifidobacterium*, *Paraprevotella* (*p* < 0.01), but also Candidatus *Stoquefichus*, *Prevotellaceae* NK3B31 group (*p* < 0.01), and Candidatus *Saccharimonas*, *Erysipelatoclostridium*, and *Odoribacter* (*p* < 0.05, Figure 5A–G, Table S1D). The post hoc Dunn’s test with Bonferroni correction revealed significant differences compared to the D-RA-NAIVE group (*p* < 0.05; Figure 5A–F, Table S1D), as well as compared to the D-RA-RELAPS group (*p* < 0.05; Figure 5A–E,G, Table S1D).

On the contrary, some genera displayed increased relative abundances in the RA-transplanted groups compared to the D-CTRL group, namely *Marvinbryantia*, *Aerococcus*, *Facklamia*, *Staphlylococcus*, *Jeotgalicoccus*, *Corynebacterium*, *Family XIII UCG-001* (*p* < 0.01), and *Caproicoproducens*, *Anaerotruncus* (*p* < 0.05; Figure 5H–P, Table S1D). The post hoc Dunn’s test showed that genera *Marvinbryantia*, *Aerococcus*, and *Facklamia* were more abundant in both RA-transplanted groups (*p* < 0.05; Figure 5H–J, Table S1D), genera *Staphlylococcus*, *Jeotgalicoccus*, *Corynebacterium*, and *Anaerotruncus* were significantly more abundant in the D-RA-RELAPS group (*p* < 0.05; Figure 5K–N, Table S1D) and the abundance of genera *Family XIII UCG-001* and *Caproicoproducens* was increased in the D-RA-NAIVE group (*p* < 0.05; Figure 5O–P, Table S1D).

Additionally, some genera reflected increased or decreased relative abundances in the D-RA-RELAPS only, as a group compared to others. These were [*Eubacterium*] nodatum group (*p* < 0.001), [*Eubacterium*] ventriosum group (*p* < 0.01), and *Sutterella*, *Fusobacterium* (*p* < 0.05; Figure 5Q–T, Table S1D). Post hoc Dunn’s test showed that genera *Sutterella* and [*Eubacterium*] nodatum group were more prevalent in the D-RA-RELAPS group compared to other groups (*p* < 0.05; Figure 5Q,R, Table S1D), and genera [*Eubacterium*] ventriosum group and *Fusobacterium* were more prevalent compared to controls (*p* < 0.05; Figure 5S–T, Table S1D). In contrast, *Tuzzerella* and [*Eubacterium*] brachy group were decreased in the relapse group (*p* < 0.05; Figure 5U–V, Table S1D). Even the group D-RA-NAIVE showed some specific genera: *Prevotellaceae UCG-001* (*p* < 0.001; Figure 5W, Table S1D) and *Helicobacter* (*p* < 0.05; Figure 5X, Table S1D).

### 2.2. Severity of Arthritis

**Figure 6:**
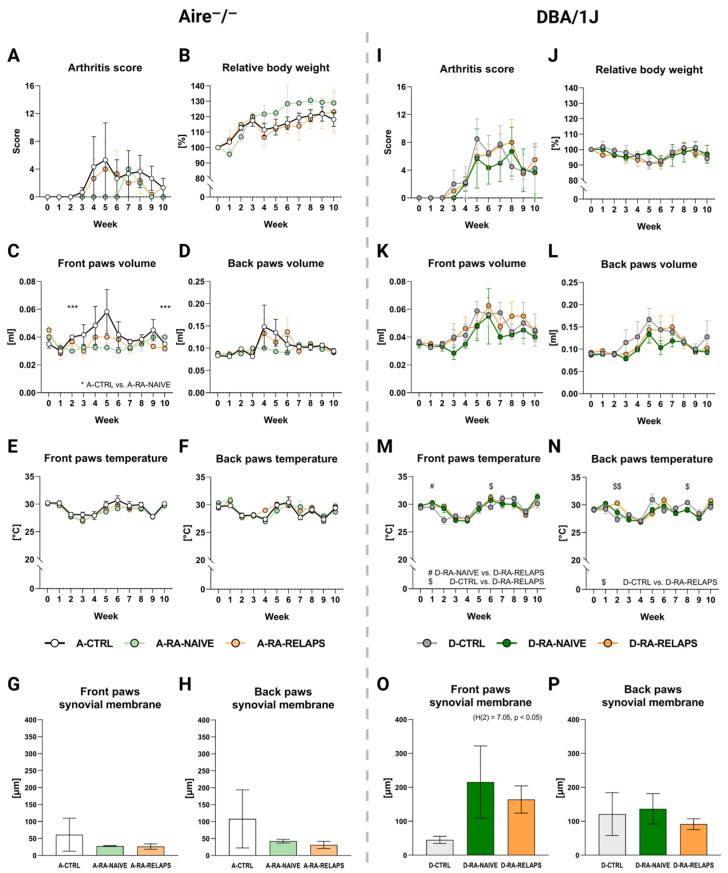
Severity of arthritis: (**A**) Dynamics of arthritis (RA) score in Aire^−^/^−^ mice, (**B**) Dynamics of relative body weight (BW) in Aire^−^/^−^ mice, (**C**) Dynamics of front paws’ volume in Aire^−^/^−^ mice, * A-CTRL vs. A-RA-NAÏVE, (**D**) Dynamics of back paws’ volume in Aire^−^/^−^ mice, (**E**) Dynamics of front paws’ temperature in Aire^−^/^−^ mice, (**F**) Dynamics of back paws’ temperature in Aire^−^/^−^ mice, (**G**) The thickness of the synovial membrane in the front paws in Aire^−^/^−^ mice, (**H**) The thickness of the synovial membrane in the back paws in Aire^−^/^−^ mice, (**I**) Dynamics of RA score in DBA/1J mice, (**J**) Dynamics of relative BW in DBA/1J mice, (**K**) Dynamics of front paws’ volume in DBA/1J mice, (**L**) Dynamics of back paws’ volume in DBA/1J mice, (**M**) Dynamics of front paws’ temperature in DBA/1J mice, (**N**) Dynamics of back paws’ temperature in DBA/1J mice, (**O**) The thickness of the synovial membrane in the front paws in DBA/1J mice, (**P**) The thickness of the synovial membrane in the back paws in DBA/1J mice. Data are presented as mean ± SEM; D-RA-NAIVE vs. D-RA-RELAPS # < 0.05, D-CTRL vs. D-RA-RELAPS $ < 0.05, $$ < 0.01, D-CTRL vs. D-RA-NAIVE *** *p* < 0.001; A-CTRL—Aire^−^/^−^ mice that received FMT from healthy donors; A-RA-NAIVE—Aire^−^/^−^ mice that received FMT from naive RA patients, A-RA-RELAPS—Aire^−^/^−^ mice that received FMT from treated RA patients in relapse; D-CTRL—DBA/1J mice that received FMT from healthy donors; D-RA-NAIVE—DBA/1J mice that received FMT from naive RA patients, D-RA-RELAPS—DBA/1J mice that received FMT from treated RA patients in relapse.

In Aire^−^/^−^ mice, no significant differences were detected in arthritis scores across groups over time (Time point: *p* = ns; group: *p* = ns; interaction: *p* = ns; Figure 6A, Table S1E). The relative body weight increased, but without significant differences across FMT-transplanted groups (Time point: *p* < 0.01; group: *p* = ns; interaction: *p* = ns; Figure 6B, Table S1E). Similarly, no FMT transplantation effect was seen on front paws’ volume (Time point: *p* = ns; group: *p* = ns; interaction: *p* = ns; Figure 6C, Table S1E) and back paws’ volume (Time point: *p* = ns; group: *p* = ns; interaction: *p* = ns; Figure 6D, Table S1E). Despite that, the post hoc Dunn’s test with Bonferroni correction revealed significant differences in front paws’ volume on weeks 2 and 10 between A-CTRL and A-RA-NAIVE groups (*p* < 0.001; Figure 6C, Table S1E). The front and back paws’ temperature increased over time but did not display any differences across FMT-transplanted groups in Aire^−^/^−^ mice (Front paws: Time point: *p* < 0.001; group: *p* = ns; interaction: *p* = ns; and back paws: time point: *p* < 0.001; group: *p* = ns; interaction: *p* = ns; Figure 6E–F, Table S1E).

To see any potential morphological changes in-colon after FMT application, the colon weight and length were measured. No significant differences were observed in the relative colon weight and colon length among Aire^−^/^−^ mice across groups at the end of the experiment (Relative colon weight: *p* = ns; colon length: *p* = ns; Table S1G).

To observe any morphological deformities in joints, such as thickening of the synovial membrane and inflammation, specifically synovial infiltration of neutrophils, histological hematoxylin and eosin (H&E) staining as well as naphthol-AS-D-chloroacetateesterase (CHAE) staining were performed. No significant differences were observed in terms of thickening of the synovial membrane among the Aire^−^/^−^ groups that had received different FMT transplants (*p* = ns; Figure 6G–H, Table S1G). A similar outcome was seen in the counts of neutrophils infiltrating synovium (*p* = ns; Figure S1A,B, Table S1G).

To see the changes in immune cells in circulation, white blood cells, lymphocytes, monocytes, and neutrophils counts were analyzed in whole blood. No significant differences were observed among Aire^−^/^−^ mice across groups at the end of the experiment (*p* = ns; Figure S2A–D, Table S1G).

In DBA/1J mice, arthritis score increased over time, but no FMT effect was observed (Time point: *p* < 0.001; group: *p* = ns; interaction: *p* = ns; Figure 6I, Table S1F). The relative body weight did not change in DBA/1J mice (Time point: *p* = ns; group: *p* = ns; interaction: *p* = ns; Figure 6J, Table S1F). The front and back paws’ volume increased over time in DBA/1J mice, but no FMT transplantation effect was seen (Front paws: time point: *p* < 0.01; group: *p* = ns; interaction: *p* = ns; and back paws: time point: *p* < 0.01; group: *p* = ns; interaction: *p* = ns; Figure 6K–L, Table S1F). The front paws’ temperature changed over time regardless of FMT transplantation (Time point: *p* < 0.001; group: *p* = ns; interaction: *p* < 0.01; Figure 6M, Table S1F). The post hoc Dunn’s test showed significant differences in weeks 1 and 6 (*p* < 0.05; Figure 6M, Table S1F). Similar results were seen in the back paws’ temperature (Time point: *p* < 0.001; group: *p* = ns; interaction: *p* < 0.001; Figure 6N, Table S1F), with the post hoc test significant differences were seen in weeks 2 and 8 (*p* < 0.05; Figure 6N, Table S1F).

FMT transplantation had no effect on the relative colon weight and colon length in DBA/1J mice (Relative colon weight: *p* = ns; colon length: *p* = ns; Table S1H).

Interestingly, the thickness of the synovium in the front paws has increased in groups that had received FMT from RA patients (*p* < 0.05; Figure 6O, Table S1H). On the contrary, no relevant differences were observed in the thickness of the back paws’ synovial membrane (*p* = ns; Figure 6P, Table S1H) and the count of neutrophils infiltrating synovium in DBA/1J mice (*p* = ns; Figure S1C,D, Table S1H).

No differences were observed in the white blood cell count, lymphocyte count, monocyte count, and neutrophil count among DBA/1J mice across groups at the end of the experiment (*p* = ns; Figure S2E,H, Table S1H).

### 2.3. Inflammatory Cytokines

**Figure 7:**
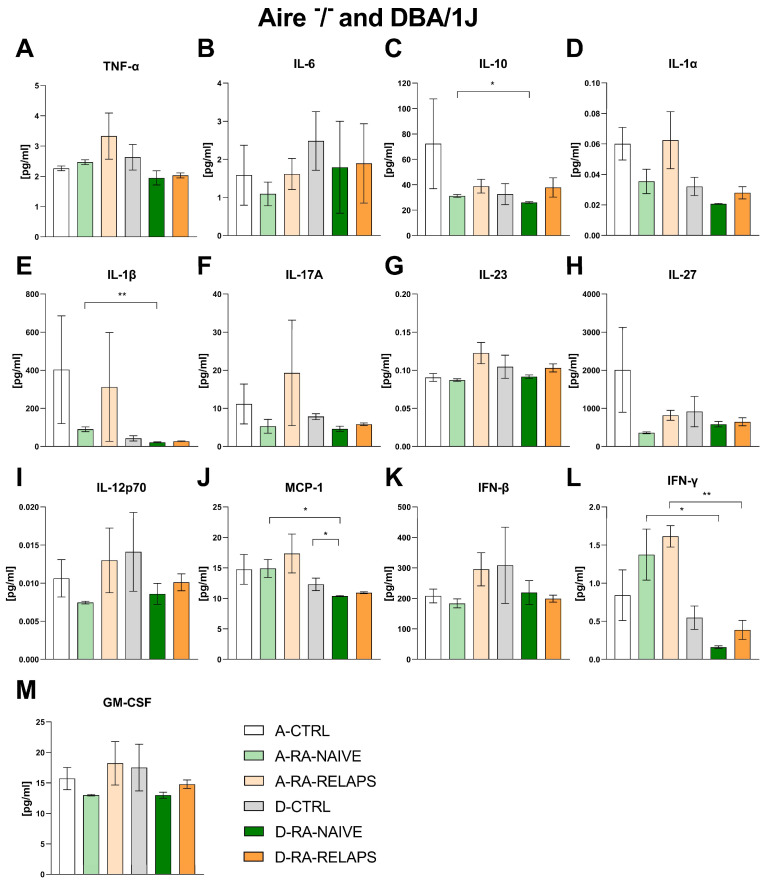
Plasma concentrations of cytokines in Aire^−^/^−^ and DBA/1J mice: (**A**) tumor necrosis factor alpha (TNF-α), (**B**) interleukin 6 (IL-6), (**C**) interleukin 10 (IL-10), (**D**) interleukin 1α (IL-1α), (**E**) interleukin 1β (IL-1β), (**F**) interleukin 17A (IL-17A), (**G**) interleukin 23 (IL-23), (**H**) interleukin 27 (IL-27), (**I**) interleukin 12p70 (IL-12p70), (**J**) monocyte chemoattractant protein 1 (MCP-1), (**K**) interferon beta (IFN-β), (**L**) interferon gamma (IFN-γ), (**M**) granulocyte-macrophage colony-stimulating factor (GM-CSF). Data are presented as mean ± SEM; * *p* < 0.05, ** *p* < 0.01; A-CTRL—Aire^−^/^−^ mice that received FMT from healthy donors; A-RA-NAIVE—Aire^−^/^−^ mice that received FMT from naive RA patients, A-RA-RELAPS—Aire^−^/^−^ mice that received FMT from treated RA patients in relapse; D-CTRL—DBA/1J mice that received FMT from healthy donors; D-RA-NAIVE—DBA/1J mice that received FMT from naive RA patients, D-RA-RELAPS—DBA/1J mice that received FMT from treated RA patients in relapse.

To evaluate potential systemic inflammatory responses, a cytokine panel was assessed in both Aire^−^/^−^ and DBA/1J mice. No differences were detected in inflammatory cytokines across different FMT groups in Aire^−^/^−^ mice, namely tumor necrosis factor alpha (TNF-α), interleukin 6 (IL-6), interleukin 10 (IL-10), interleukin 1α (IL-1α), interleukin 1β (IL-1β), interleukin 17A (IL-17A), interleukin 23 (IL-23), interleukin 27 (IL-27), interleukin 12p70 (IL-12p70), monocyte chemoattractant protein 1 (MCP-1), interferon beta (IFN-β), interferon gamma (IFN-γ), and granulocyte-macrophage colony-stimulating factor (GM-CSF), (*p* = ns; Figure 7A–M, Table S1K). The same outcome was observed in DBA/1J mice that received transplants from different donors, except for MCP-1 (*p* = ns; Figure 7A–I, 7K–M, Table S1L). The post hoc Dunn’s test with Bonferroni correction detected decreased MCP-1 in the D-RA-NAIVE group compared to controls (*p* < 0.05; Figure 7J). Interestingly, we detected strain-specific differences in groups that received the same FMT transplants. Namely, IL-10, MCP-1, IFN-γ (*p* < 0.05; Figure 7C,J,L), and IL-1β (*p* < 0.01; Figure 7E) differed across A-RA-NAIVE and D-RA-NAIVE groups, while IFN-γ differed across A-RA-RELAPS and D-RA-RELAPS groups (*p* < 0.05; Figure 7L, Table S1M).

### 2.4. Correlation Severity of Arthritis and Gut Microbiota

**Figure 8:**
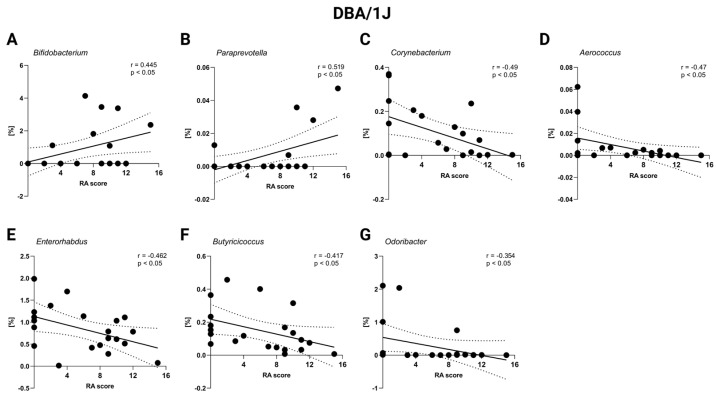
Correlation of RA score and relative abundance of specific bacteria in DBA/1J mice: (**A**) *Bifidobacterium*, (**B**) *Paraprevotella*, (**C**) *Corynebacterium*, (**D**) *Aerococcus*, (**E**) *Enterorhabdus*, (**F**) *Butyricoccus*, and (**G**) *Odoribacter*. Dot—mouse recipient samples; x axis—Artritic score (RA) score; y axis—relative abundance in %; solid line—best-fit linear regression line; dashed line—95% confidence interval; presented data show DBA/1J mice from all groups: D-CTRL, D-RA-NAIVE, and D-RA-RELAPS.

Lastly, to investigate the relationship between gut microbiota composition and arthritis severity, the correlations between RA scores and the relative abundance of specific bacterial genera were analyzed. No significant correlations were detected in Aire^−^/^−^ mice (*p* = ns, Table S1I). However, a significant positive correlation was observed between *Bifidobacterium* and RA score in DBA/1J mice (*p* < 0.05; Figure 8A, Table S1J). Similarly, genus *Paraprevotella* also exhibited a significant positive correlation with RA score (*p* < 0.05; Figure 8B, Table S1J), indicating a potential association with RA progression. Conversely, *Corynebacterium*, *Aeroccocus, Enterorhabdus*, *Butyricicoccus*, and *Odoribacter* all displayed significant negative correlations with RA score in DBA/1J mice, suggesting a potential protective role of these genera in RA pathogenesis (*p* < 0.05; Figure 8C–G, Table S1J.)

**Figure 9:**
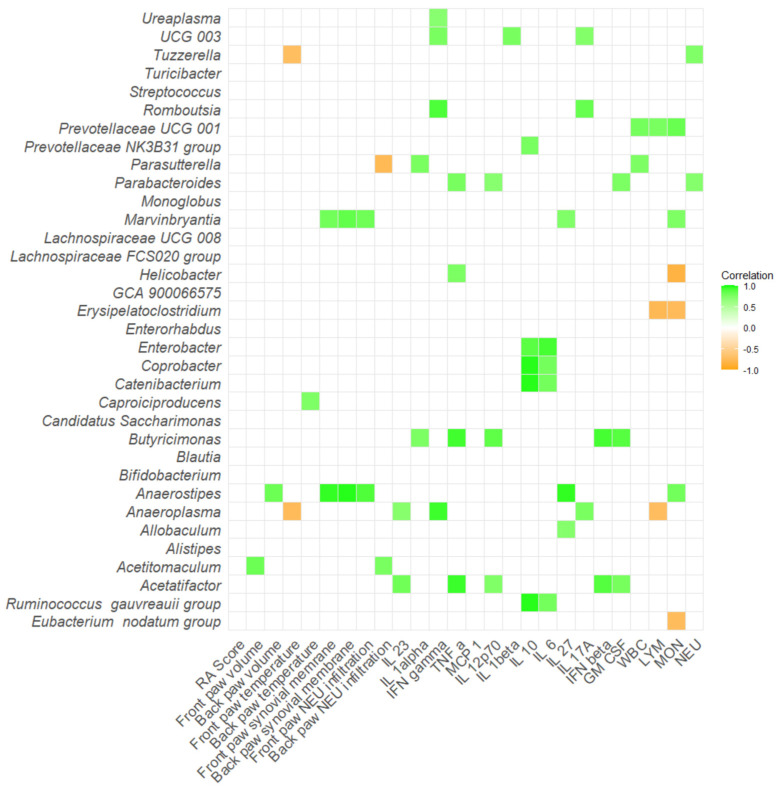
Correlation matrix of severity of arthritis, inflammatory cytokines and relative abundance of specific bacteria in Aire^−^/^−^ mice. All data presented are analyzed at the genus level with *p* < 0.05. RA score—Artritic score; IL 23—interleukin 23; IL 1alpha—interleukin 1α; IFN gamma—interferon γ; TNF alpha—tumor necrosis factor α; MCP 1—monocyte chemoattractant protein 1; IL 12p70—interleukin 12p70; IL 1beta—interleukin 1β; IL 10—interleukin 10; IL 6—interleukin 6; IL 27—interleukin 27; IL 17A—interleukin 17A; IFN beta—interferon β; GM CSF—granulocyte-macrophage colony-stimulating factor; WBC—white blood cells; LYM—lymphocytes; MON—monocytes; NEU—neutrophils.

Additionally, a correlation matrix between the severity of arthritis, inflammatory cytokines, versus relative abundance of microbial taxa that differed across the groups was performed. In Aire^−^/^−^ mice, bacteria such as *Butyricimonas* and *Acetatifactor* positively correlated with numerous proinflammatory cytokines and molecules, namely TNF-α, IL-12p70, IFN-β, and GM-CSF (*p* < 0.05; Figure 9, Table S1I). Numerous bacteria positively correlated with IL-10 and IL-6, including, *Enterobacter*, *Coprobacter*, *Catenibacterium*, and [*Ruminococcus*] gauvreauii group (*p* < 0.05; Figure 9, Table S1I). Interestingly, *Anaerostipes* positively correlated with paw temperature, increased thickness of synovial membrane, and neutrophil infiltration in joints (*p* < 0.05; Figure 9, Table S1I).

**Figure 10:**
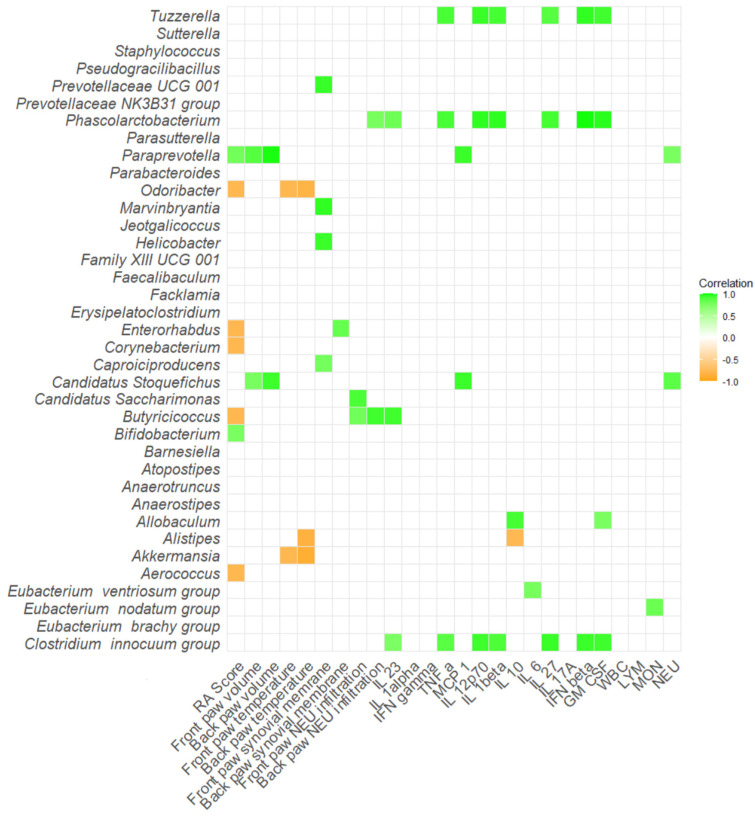
Correlation matrix of severity of arthritis, inflammatory cytokines and relative abundance of specific bacteria in DBA/1J mice. All data presented are analyzed at the genus level with * *p* < 0.05. RA score—Artritic score; IL 23—interleukin 23; IL 1alpha—interleukin 1α; IFN gamma—interferon γ; TNF alpha—tumor necrosis factor α; MCP 1—monocyte chemoattractant protein 1; IL 12p70—interleukin 12p70; IL 1beta—interleukin 1β; IL 10—interleukin 10; IL 6—interleukin 6; IL 27—interleukin 27; IL 17A—interleukin 17A; IFN beta—interferon β; GM CSF—granulocyte-macrophage colony-stimulating factor; WBC—white blood cells; LYM—lymphocytes; MON—monocytes; NEU—neutrophils.

Several bacteria showed positive correlations with proinflammatory cytokines and molecules in DBA/1J mice as well. For instance, bacteria *Tuzzerella*, *Phascolarctobacterium*, *Allobaculum* and [*Clostridium*] innocuum group positively correlated with at least two of following: IL-23, TNF-α, IL-12p70, IL-1β, IL-27, IFN-β, or GM-CSF (*p* < 0.05; Figure 10, Table S1J). Interestingly, *Paraprevotella* showed positive correlations with not only RA score, but also increased paw swelling in both front and back paws (*p* < 0.05; Figure 10, Table S1J). On the contrary, bacterium *Odoribacter* showed negative correlations with paw temperature alongside the negatively associated RA score (*p* < 0.05; Figure 10, Table S1J).

## 3. Discussion

The main aim of this study was to explore the role of gut microbiota in the onset and progression of RA using the CIA model combined with the FMT method. The CIA represents a widely applied and standardized model in the preclinical research of RA. It is induced in genetically susceptible mice by immunization with type II heterologous collagen emulsified in Freund’s adjuvant, leading to pathological characteristics similar to RA, such as mononuclear cell infiltration or cartilage destruction [24,29]. In this article, two different mouse strains were used to analyze the role of the gut microbiota in the CIA model. While the DBA/1J mice are widely used in the CIA, as shown by other studies [25,26,27], the application of the Aire^−^/^−^ mouse strain represents a novel strategy in studying the pathogenesis of RA. The transcription factor AIRE is crucial for preventing autoimmunity. As shown in Aire^−^/^−^ mice, its absence leads to autoantibody production and a variety of immune system defects [28]. So far, Aire^−^/^−^ mice have been utilized in animal studies of autoimmune-mediated exocrinopathy resembling the clinical symptoms of peripheral neuropathy associated with Sjögren’s syndrome [30], polyglandular autoimmune syndrome type 1 [31], autoimmune uveitis [32], and autoimmune hepatitis [33], but experimental studies on RA are lacking. However, studies from Spanish and Japanese populations suggest the association of the *AIRE* gene and susceptibility to RA [34,35]. The expression of *AIRE* in CD14+ primary human mononuclear cells cultures showed a significant decrease in RA patients compared to healthy subjects. Moreover, this resulted in the differentiation of T follicular helper cells [36]. This evidence suggests that the onset of RA could be related to the decreased expression of *AIRE*. Therefore, incorporating the Aire^−^/^−^ mice could represent an interesting model for investigating RA pathogenesis. Two different sources of type II collagen, based on the different susceptibility of the mouse strains, were used. DBA/1J mice, which are highly susceptible to CIA, received bovine collagen. In contrast, AIRE^−^/^−^ mice are on the C57BL/6 background, known to be less susceptible [37]. Therefore, these mice were given chicken collagen, as recommended for low-susceptibility strains [38]. This approach ensured effective and strain-appropriate induction of arthritis, consistent with established protocols and literature.

Recent research suggests that gut microbiota may play a role in the development or the progression of numerous autoimmune diseases [39]. Clinical studies highlight the change in gut microbiota composition in RA patients as well [40,41]. The sequencing of the V3-V4 variable regions of bacterial 16S rRNA genes revealed an increased abundance of *Escherichia-Shigella* but decreased abundances of *Alloprevotella*, *Lactobacillus*, *Enterobacter*, and *Odoribacter* in Chinese RA patients, compared to healthy individuals [40]. Similarly, the sequencing of the V4 region of the 16S rRNA gene showed increased abundance of *Lachnospiraceae*, *Helicobacteraceae*, *Ruminococcaceae*, *Erysipelotrichaceae*, and *Bifidobacteriaceae* in anti-CCP positive individuals without clinical synovitis, but subsequently 20% of individuals progressed to RA [41]. Therefore, therapeutic strategies aiming at modulating gut microbiota composition hold potential for treating these conditions. The FMT method has been shown to restore dysbiotic gut microbiota, reestablish intestinal homeostasis, and both innate and adaptive immune responses [42].

In this study, FMT pools from three different groups: healthy individuals, naive RA patients, and treated RA patients in relapse, were continuously transplanted once a week during the induction of the CIA model (Figure 9). Initial PCA analysis showed very conserved clustering patterns of samples before and after FMT (Figure 1A), suggesting the shift of gut microbiota composition after transplantation and successful FMT procedure. This is supported by RDA analysis as well, where donors’ pools and corresponding recipients’ mouse samples were clustered alike (Figure 1E,H). In contrast, no differences were detected across different time points after FMT, week 5 and week 10, leading to pooled sequencing analysis. Interestingly, the results show strain-specific colonization from the same FMT donors, suggesting that the choice of mouse strain in FMT can play a role in experimental FMT studies. Despite cluster overlap in PCA analysis across groups in individual mouse strains (Figure 1D,G), the RDA analysis showed a particular separation of samples after receiving FMT from different donors (Figure 1E,H).

Subsequent analyses showed several specific genera for CTRL-pool as well as RA-RELAPS-pool transplanted mice. Regardless of the strain of mice, all analyses detected an increased relative abundance of *Bifidobacterium* in CTRL-pool transplanted mice. This genus is a common probiotic bacterium that is oftentimes found in probiotic-enriched dairy products, probiotic supplements, and the healthy gastrointestinal tract [43]. Surprisingly, the A-CTRL group was enriched in *Paraprevotella*, *Prevotellaceae* UCG-001, and *Parabacteroides* in Aire^−^/^−^ mice as well. The current clinical studies suggest the causal role of these bacteria in the onset of RA. Enrichment of the family *Prevotellaceae* and genus *Prevotella* was detected in the pre-clinical RA patients [17]. Similarly, *Parabacteroides*, *Paraprevotella*, but also genera *Bacteroides*, *Porphyromonadaceae*, *Phascolarctobacterium*, and *Carnobacterium* were enriched in naive RA patients [44]. On the other hand, butyrate-producing bacteria, including *Faecalibacterium*, *Roseburia*, *Subdoligranulum*, *Ruminococcus*, and *Pseudobutyrivibrio*, were reduced in RA patients while being more abundant in healthy individuals [44]. In our study, these bacteria were not enriched in the CTRL-transplanted mice. Controversially, *Prevotella* could not be necessarily harmful; in fact, some studies suggest it may have beneficial effects in certain contexts. An animal study of arthritis-susceptible HLA-DQ8 mice immunized with type II collagen exhibited significantly decreased incidence and severity of arthritis compared to controls after treatment with the commensal bacterium *Prevotella histicola* [45].

Usually, the first signs of arthritis seems to appear 3–4 weeks after immunization [25]. In our experimental settings, we observed the first arthritis score since week 3, as expected. Except for the thickening of the front paws’ synovial membrane, there were minor differences in arthritis score, relative body weight, paw volume, paw temperature, neutrophilic synovial infiltrations across different FMT-transplanted groups. Similarly, minor differences were detected in systemic inflammatory responses, except for MCP-1. This protein contributes to joint damage by recruiting monocytes and promoting inflammation [46]. Studies show that the secretion of MCP-1 could be indirectly linked to gut microbiota. A high-fiber dietary intervention in RA patients led to an increase in short-chain fatty acids and a shift in the *Firmicutes*-to-*Bacteroidetes* ratio resulting in a reduction in proinflammatory cytokines, including decreased secretion of the MCP-1, but also interleukin-18 and interleukin-33 [47]. The unexpected weight gain in arthritic Aire^−^/^−^ mice may arise from a combination of immune system dysregulation and altered gut microbiota composition. Impaired central immune tolerance and systemic autoimmunity could interact differently with the transplanted microbiota and affect the production of short-chain fatty acids (SCFAs), like propionate and butyrate. These SCFAs play key roles in lipid metabolism, insulin sensitivity, and energy storage through G-protein-coupled receptors, potentially promoting increased fat accumulation [48]. The observed changes in cytokine concentrations between Aire^−^/^−^ and DBA/1J mice were expected, reflecting Aire-deficient mice’s known immune dysregulation profile.

We detected several correlations that may indicate the potential role of some bacterial taxa in the pathogenesis of RA. Surprisingly, a positive correlation between RA score and genus *Bifidobacterium* was detected in DBA/1J mice, which is in contrast to already published data. In an intervention study, the supplementation of different strains of *Bifidobacterium adolescentis*, in the form of oral gavage, led to an alleviation of CIA symptoms and a restoration of proinflammatory and anti-inflammatory responses in the CIA model rats. Interestingly, an early administration of *B. adolescentis* before inducing the CIA model led to a better outcome than late *B. adolescentis* treatment [49]. Also, intervention with *Bifidobacterium longum* RAPO in CIA mice ameliorated inflammation, as shown by reduced arthritis score, bone damage, and cartilage damage, supposedly by inhibiting the secretion of IL-17 and other proinflammatory mediators [50]. Similarly, as already published studies have observed, a positive correlation between RA score and *Paraprevotella* was shown. Despite a majority of studies focusing on the genus *Prevotella* as an RA risk factor in the preclinical RA stage [51], *Paraprevotella* is reported to be enriched in RA patients as well [44].

Three bacteria, *Odoribacter*, *Enterorhabdus* and *Butyricicoccus*, showed negative correlations with RA score in this study, indicating their potential positive effect. In the mouse model of CIA, *Enterorhabdus* was significantly less abundant in CIA-susceptible mice prior to arthritis onset compared to CIA-resistant animals [52]. Interestingly, *Enterorhabdus*, but also other genera, including *Myroides*, *Rikenella*, *Brochothrix*, *Lactococcus*, and *Streptococcus*, were less abundant in arthritic mice when compared with untreated mice. Contrarily, *Odoribacter*, but also *Desulfovibrio*, *Prevotella*, *Parabacteroides*, *Acetatifactor*, *Blautia*, *Coprococcus*, and *Ruminococcus* were more abundant in arthritic mice [52].

The absence of significant correlations between specific bacterial taxa and arthritis scores in Aire^−^/^−^ mice could reflect both immunological and microbiota differences, as described above. Moreover, DBA/1J mice, which are more susceptible to the CIA model, developed more severe and consistent arthritis, allowing for clearer microbiota–disease associations. In contrast, Aire^−^/^−^ mice exhibited milder, more variable signs of arthritis, reducing the statistical power to detect such correlations.

Additionally, we performed a correlation analysis of numerous other parameters, including paw swelling and temperature of inflammatory cytokines, with the relative abundances of significantly different genera, and observed minor yet relevant observations. Clinical studies have already reported positive correlations with biomarkers of inflammation. For instance, genera *Dorea* and *Ruminococcus* showed a positive correlation with RF-IgA and anti-CCP antibodies, *Prevotella*-2 and *Alloprevotella* with C-reactive protein, and *Alloprevotella* and *Parabacteroides* with the erythrocyte sedimentation rate [40]. Interleukins such as IL-23, TNF-α, IL-1β, GM-CSF, and IL-10 are critical mediators in the pathogenesis of RA, contributing to either proinflammatory responses and joint destruction or to the modulation of immune regulation [53]. In our correlation analysis using the CIA model in DBA/1J mice following FMT, we observed significant positive associations between these interleukins and specific microbial taxa. In DBA/1J mice, known for their susceptibility to arthritis, we identified a positive correlation between *Paraprevotella* abundance and RA severity, as observed above, but also with paw swelling. This finding supports previous observations that *Paraprevotella* is enriched in the gut microbiota of RA patients [54]. Notably, *Allobaculum* was identified as a differentially abundant genus in RA-associated microbiota [55]. Similarly, *Phascolarctobacterium* has been previously implicated in inflammatory conditions such as rheumatoid arthritis [44], suggesting a broader role in inflammation. Intriguing results were also observed with *Tuzzerella*. Although information on this genus in the context of RA is limited, it has been described in mouse models of severe acute malnutrition [56], warranting further investigation. *Clostridium*-like bacteria, particularly *Clostridium innocuum*, were found to be more prevalent in the gut microbiota of HLA-DRB1*0401 transgenic mice, which are susceptible to arthritis. This correlation highlights the potential role of specific gut microbial taxa in modulating immune responses and underscores the significance of the gut–joint axis in RA pathophysiology [57]. In contrast, *Odoribacter* demonstrated anti-inflammatory associations, as reflected by its negative correlation with the RA score. These properties have also been documented in other inflammatory conditions such as inflammatory bowel disease [58]. In Aire^−^/^−^ mice, PCA revealed a distinct microbiome composition compared to DBA/1J mice. This divergence was also reflected in the correlation matrix, where genera such as *Butyricimonas* and *Acetatifactor* were positively associated with proinflammatory cytokines in the CIA model, aligning with previously published findings [59,60]. Lastly, *Anaerostipes* showed a positive correlation with the anti-inflammatory cytokine IL-27 [61], consistent with the prior literature where this genus was inversely associated with clinical severity in autoinflammatory diseases [62].

The results suggest that the gut microbiota pool from healthy individuals might contain species profoundly connected to RA onset, strongly affecting the final outcome, resulting in no significant differences across groups. This data holds potential for further investigation of these bacteria in the pathogenesis of RA. Application of pooled fecal samples from different donors enhances the overall diversity of microbial communities. In our study, three donor samples per group were pooled before transplantation. The number of donors used in our study aligns with established practices in FMT research. Most clinical studies typically utilize a single donor, with pooling limited to a maximum of five donors [63,64]. Similarly, experimental studies use samples from multiple time points of a single donor or pool samples from up to five donors [65,66].

Despite mainly negative results, this study provides novel insights and offers a valuable perspective. Unlike observational human studies, which can only identify associations, FMT allows direct manipulation of the gut microbiota in a controlled setting. A well-defined animal model, such as DBA/1J mice, provides a standardized approach for studying disease severity. Subsequently, the use of the Aire^−^/^−^ mouse strain, which has not been employed in the CIA model so far, represents the novelty of this study. The application of FMT, as an emerging approach for understanding autoimmune diseases, could elucidate the role of specific bacterial populations in the onset and progression of RA. Simultaneously, it could serve as a new personalized therapeutic tool in the future.

In contrast, one of the limitations of this study is the relatively low sample size per group, which may represent a challenge when analyzing the microbiome. Given the complexity of microbial communities and the presence of numerous low-abundance bacterial species, a larger cohort would enhance the statistical power and reliability of these findings. Another limitation is the application of the variable V4 region of the 16S rRNA amplicon sequencing rather than whole metagenome sequencing, which restricts the resolution of microbial analysis only to genus and prevents functional characterization of the microbiome. Finally, the absence of control groups, sham-FMT, or groups without RA induction limits the ability to fully unravel the specific effects of FMT and gut microbiota on the severity of arthritis.

Despite minor differences observed in the severity of the arthritis across groups that had received different FMTs, correlations between the abundance of specific bacterial strains and RA score were detected. Future studies incorporating larger sample sizes, more comprehensive sequencing approaches, and additional control groups would strengthen the interpretability of the results. Yet, insights gained from this preliminary study may provide valuable information for microbiome-targeted therapies, offering new approaches to autoimmune diseases in the future.

## 4. Materials and Methods

### 4.1. Donor Sample Collection

Stool samples were collected from RA donors and healthy age- and sex-matched controls. The RA donors were diagnosed according to the European Alliance of Associations for Rheumatology (EULAR) criteria [67] and were recruited in collaboration with the 1st Department of Internal Medicine at the University Hospital, Faculty of Medicine, Comenius University in Bratislava, and the Department of Rheumatology at Saint Michael’s Hospital, Bratislava, Slovakia. The inclusion criteria for RA donors were as follows: RA patients who were either treatment-naïve or in the relapse phase while receiving methotrexate, disease duration of no more than five years, female sex, and a minimum age of 18 years. Exclusion criteria for all donors included antibiotic treatment in the past three months, excessive alcohol consumption in the past month, acute inflammation at the time of stool collection, chronic clinically relevant digestive tract diseases (e.g., ulcerative colitis, Crohn’s disease), HIV, HBV, HCV, suspected toxic shock, or any other autoimmune diseases. Relevant clinical data, according to the EULAR classification criteria, including disease activity score-28 (DAS28), anti-cyclic citrullinated peptide antibodies (Anti-CCP), RF, C-reactive protein (CRP), erythrocyte sedimentation rate (ESR), lymphocyte count (LYM), leukocyte count (LEU), monocyte count (MON), as well as patient age and RA duration, are summarized in Table 1. Samples were collected using sterile, leak-proof containers, and participants were instructed to avoid contamination. The collected stool samples were stored at 4 °C immediately after collection, transported to the laboratory, and processed for further analysis within two hours.

### 4.2. Recipient Housing Conditions

In this study, 20 female adult mice were used: DBA/1J mice (*n* = 11, Strain #000670, Jackson Laboratory, Bar Harbor, ME, USA) and B6.129S2-Airetm1.1Doi/J syn. (Aire^−^/^−^ mice, *n* = 9, Strain #004743, Jackson Laboratory, Bar Harbor, ME, USA). Experimental animals were group-housed under standard conditions in individually ventilated cages, maintained at a temperature of 22 ± 2 °C, a humidity of 50 ± 10%, and a 12-h light–dark cycle. Animals had free access to standard chow (KMK20, EYPY, Czech Republic) and tap water *ad libitum*. The experiment was conducted in accordance with the Animal Research: Reporting of In Vivo Experiments (ARRIVE 2.0) guidelines.

### 4.3. Experimental Groups and Study Design

**Figure 11:**
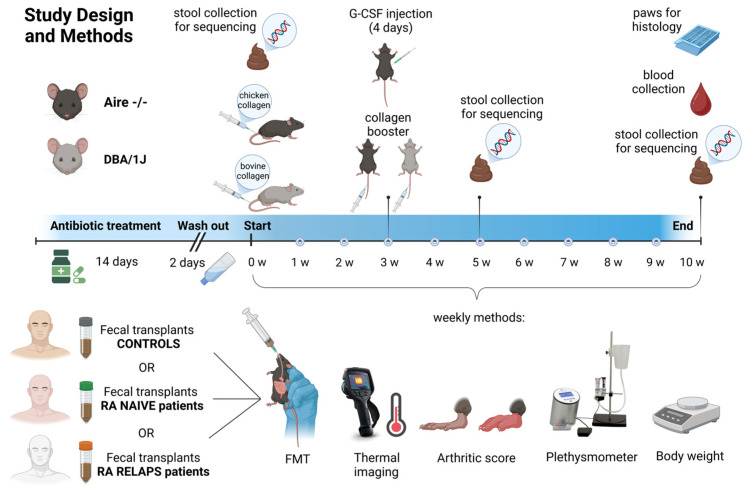
Study design and methods. Two mouse strains, Aire^−^/^−^ and DBA/1J, underwent antibiotic treatment for 14 days, followed by a 2-day washout period. Subsequently, each mouse strain was divided into three subgroups, based on different fecal microbiota transplantation (FMT) to be received, and a collagen antibody-induced arthritis (CIA) model was induced in all groups for 10 weeks. CIA in DBA/IJ mice was induced using bovine type II collagen, while chicken type II collagen was used in Aire^−^/^−^ mice. Once a week, FMT from the respective donors was received, accompanied by thermal imaging, arthritic score, plethysmometer, and bodyweight measurements. Fecal samples of recipients were collected at three time points and were sequenced. RA—rheumatoid artritis; w—week; G-CSF—Granulocyte colony stimulating factor.

Both mouse strains, Aire^−^/^−^ and DBA/1J, underwent a 14-day antibiotic treatment, followed by a 2-day washout period. The antibiotic cocktail consisted of vancomycin (500 mg/L), ampicillin (1 g/L), metronidazole (1 g/L), and neomycin (1 g/L) provided in the tap water. Subsequently, each mouse strain was subdivided into three experimental groups, with all groups sharing the CIA model (Figure 11). For DBA/IJ mice, CIA induction was carried out via an intradermal injection of 100 μL of a 1:1 emulsion containing bovine type II collagen (CII; Chondrex, Redmond-Woodinville, WA, USA) and complete Freund’s adjuvant (CFA; Chondrex, Redmond-Woodinville, WA, USA), which included Mycobacterium tuberculosis at a concentration of 2 mg/mL. This emulsion was administered subcutaneously at the base of the tail, following the procedure outlined by [24]. The protocol was identical for Aire^−^/^−^ mice, except that chicken type II collagen (Chondrex, Redmond-Woodinville, WA, USA) was used in place of bovine collagen. Both mouse strains received a booster injection of the appropriate collagen, based on the Chondrex protocol, on day 21 of the experiment [37]. Additionally, Aire^−^/^−^ mice were treated subcutaneously with granulocyte colony-stimulating factor (G-CSF) for four consecutive days. Aire^−^/^−^ mice were divided into a group that followed the CIA induction protocol and received FMT from healthy donors (A-CTRL; *n* = 3), a group that followed the same protocol and received FMT from naive RA patients (A-RA-NAIVE; *n* = 3), and a group that followed the same protocol and received FMT from treated RA patients in relapse (A-RA-RELAPS; *n* = 3; Figure 11). Similarly, DBA/1J mice were divided into a group that followed the CIA induction protocol and received FMT from healthy donors (D-CTRL; *n* = 4), a group that followed the same protocol and received FMT from naive RA patients (D-RA-NAIVE; *n* = 3), and a group that followed the same protocol and received FMT from treated RA patients in relapse (D-RA-RELAPS; *n* = 4; Figure 11).

### 4.4. Fecal Filtrate Preparation and Fecal Microbiota Transplantation

Up to 30 g of freshly collected donor fecal sample was four-times diluted and homogenized in 0.9% sterile saline solution (B. Braun Melsungen AG, Melsungen, Germany). Large particles were removed with centrifugation at 400 g for 10 min at 4 °C. The supernatant was aliquoted into individual dosages for each week separately to avoid repeated defrosting of samples. Fecal filtrates were stored in sterile glycerol (Sigma, St Louis, MO, USA) to a final concentration of 10% and frozen at −80 °C. On the day of FMT administration, aliquots were defrosted in a water bath at 37 °C. FMT was administered by oral gavage in a volume of 250 µL per animal once a week for 10 weeks (Figure 9).

### 4.5. Recipient Sample Collection

Stool samples from mouse recipients were collected once a week, before FMT administration, by abdominal massage into sterile 1.5 mL Eppendorf tubes and immediately stored at −80 °C until further microbiome analysis (Figure 9). At the end of the experiment, animals were anesthetized with inhalation anesthesia of 3% isoflurane (Vetpharma Animal Health, Barcelona, Spain) and blood was collected from the retro-orbital plexus into the EDTA collection tubes (Sarstedt, Nümbrecht, Germany). Blood count was analyzed using the hemoanalyzer Abacus VET 5 (Diatron MI ZRT., Budapest, Hungary). Plasma was centrifuged at 1600 g for 10 min at 4 °C and stored until further analysis at −20 °C. Front and back paws were collected for histology analysis, and relative colon weight and colon length were measured at the end of the experiment.

### 4.6. Library Preparation and Sequencing

Genomic DNA from donors’ and recipients’ samples was extracted using a commercial QIAamp Fast DNA Stool Mini kit (Qiagen, Hilden, Germany) according to the manufacturer’s instructions. The DNA quality and quantity were verified by a spectrophotometric method using the Qubit dsDNA HS Assay Kit (Thermo Fisher Scientific Inc., Waltham, MA, USA). The variable V4 region of the 16S rRNA gene was amplified, and samples were pair-end sequenced on the MiSeq Sequencing System of the Illumina platform (ID M07930) at the BioVendor MDx, Brno, Czech Republic.

### 4.7. Bioinformatic Analysis

The quality of raw fastq sequences was assessed using tools FastQC (version 0.11.9) [68] and MultiQC (version 1.13) [69]. To remove low-quality reads, Fastp (version 0.23.2) [70] was utilized. The filtering criteria required a minimum read length of 50 bases. Sequences were processed from the 3’ to the 5’ end using a sliding window approach of 10 bases and a mean quality score of at least 30. Primer sequences were removed with Cutadapt (version 3.5) [71], applying the linked behavior and overlap threshold of 10 nucleotides. Untrimmed sequences were discarded from the analysis. Data passing the quality filtering and primer trimming were processed using the DADA2 (version 1.22.0) [72] bioinformatics pipeline and software package. The package was employed for ASV generation and chimera removal. The forward and reverse reads were merged using a minimum overlap of 20 bp. Finally, the 16S rRNA V4 gene sequences were classified using the IDTAXA classifier in the DECIPHER (version 2.22.0) [73] package, with a minimum confidence threshold of 50%, and the SILVA SSU r138 reference database. The final ASV table was used for downstream diversity and statistical analyses.

### 4.8. Severity of Arthritis and Paw Swelling

The severity of arthritis was visually evaluated using a scoring system ranging from 0 to 4 points for each paw separately [37]. For scoring, three types of joints were observed: interphalangeal, metacarpophalangeal, and carpal for the front paws or tarsal for the back paws. The score was as follows: 0 points stand for the physiologic state; 1 point represents the redness and swelling of one of the three above-mentioned joint types; 2 points represent the redness and swelling of two of the three above-mentioned joint types; 3 points represent the redness and swelling of all three above-mentioned joint types; and, lastly, 4 point shows the achievement of maximal redness and swelling of the entire paw. Paw swelling was quantitatively assessed using a digital plethysmometer (Ugo Basile, Comerio VA, Italy). The front paws were immersed in the liquid up to the level of the carpal joint, while the back paws were immersed up to the level of the tarsal joint [25]. The technical variability of the plethysmometer measurements was less than 5%.

### 4.9. Paw Temperature

Infrared thermography was employed to evaluate the severity of paw inflammation based on temperature. Thermal images of all four paws were obtained using a thermal camera (Teledyne FLIR-E64501, Wilsonville, OR. United States) connected to the computer. To minimize the effect of the surrounding environment, the measurements were conducted in a room with a controlled temperature maintained at 25 °C, and the mice were adapted to the room conditions 30 min before the thermal imaging. Mice were then placed into an induction chamber with a continuous flow of isoflurane (3%) (Vetpharma Animal Health, Barcelona, Spain) mixed with oxygen (97%). The camera was adjusted to keep a constant distance of 20 cm from the mouse. To avoid direct contact with the mouse body, mice were handled only by touching the tip of their tail. Mice were positioned in a prone posture, with the front and hind legs extended away from the body using tweezers. The captured thermal images were analyzed using the FLIR Tools software (version 5.x). To determine the temperature of the paws, elliptical regions of interest (ROI) were placed over the paws of the mice. ROIs were sized identically for the entire study.

### 4.10. Histology

Paw tissue samples were collected and fixed in 4% formaldehyde, embedded in paraffin, and cut in 4 μm thick slices. Samples were stained with H&E for analysis of the thickness of the synovial membrane and CHAE activity for the detection of tissue infiltration by neutrophils. Samples were evaluated by light microscopy (LeicaDM2000, Wetzlar, Germany). The thickness of the synovial membrane was calculated as an average of three representative cartilage areas per mouse for each front and back paw separately. For calculation of infiltrating neutrophils, the number of cells localized around the joint was calculated as an average of three representative cartilage areas per mouse, for front and back paws separately, as well.

### 4.11. Inflammatory Cytokines Concentration

Circulating concentrations of inflammatory cytokines, including TNFα, IL-1α, IL-10, IL-6, IL-1β, IL-23, IFN-γ, IFN-β, GM-CSF, IL-17A, IL-27, and MCP-1, were quantified using the LEGENDplex™ Mouse Inflammation Panel (BioLegend, San Diego, CA, USA). The assay was performed following the manufacturer’s protocol. Samples were analyzed using a DxFLEX flow cytometer (Beckman Coulter Life Sciences, Indianapolis, IN, USA). Cytokine concentrations were determined by interpolation from standard curves generated with known concentrations of the respective cytokines.

### 4.12. Statistical Analysis

Statistical evaluation and data visualization of arthritis score, body weight, plethysmometry, temperature, colon weight and length, and blood parameters were performed using the GraphPad Prism software (version 8.0.10; GraphPad Software, Inc., San Diego, CA, USA). RStudio (version 4.2.1) served as the primary software tool to analyze the microbiome data. ASVs with less than one count were removed, and the data were normalized. The PCA analysis, RDA analysis, Shannon diversity index and correlation matrix were obtained using vegan and ggplot2 libraries. Differences across the groups were analyzed with one-way ANOVA and Bonferroni post hoc tests. Differences were considered statistically significant if *p*-values were less than 0.05, as shown by asterisks (* *p* < 0.05, ** *p* < 0.01, *** *p* < 0.001). The presented results were visualized as an average of ± standard error of the mean. RDA was performed with 1000 permutation tests. Schemes were created with Biorender.com.

## 5. Conclusions

This study provides important insights into the relationship between gut microbiota and RA, highlighting both potentially pathogenic and protective microbial genera. Although no direct effects of FMT on arthritis severity were demonstrated, the observed microbiota alterations underscore the complexity of host–microbe interactions in autoimmune diseases. Future research with expanded sample sizes, advanced microbiome analysis methods, and additional experimental controls will be crucial to clarify these interactions and validate the microbiome as a therapeutic target in RA management.

## Figures and Tables

**Table 1:** Donor clinical data: naive rheumatoid arthritis (RA) patients (RA-NAIVE), treated RA patients in relapse (RA-RELAPS), disease activity score-28 (DAS28), anti-cyclic citrullinated peptide antibodies (Anti-CCP), rheumatoid factor (RF), C-reactive protein (CRP), erythrocyte sedimentation rate (ESR), lymphocyte count (LYM), leukocyte count (LEU), monocyte count (MON).

	RA-NAIVE	RA-RELAPS	*p*-Value
Age	55.67 ± 8.45	59.25 ± 7.59	ns
Duration of RA	0.75 ± 0.75	4.67 ± 0.17	<0.01
DAS28	4.83 ± 0.78	4.99 ± 0.44	ns
Anti-CCP [U/mL]	21.80 ± 18.17	121.70 ± 49.17	ns
RF [IU/mL]	11.00 ± 1.00	15.00 ± 2.08	ns
CRP [mg/L]	29.95 ± 27.03	25.63 ± 22.24	ns
ESR [mm/h.]	34.50 ± 24.10	55.67 ± 15.51	ns
LYM [x 10⁹/l]	1.840 ± 0.20	1.62 ± 0.39	ns
LEU [× 10⁹/l]	7.62 ± 0.91	5.56 ± 0.97	ns
NEU [× 10⁹/l]	5.17 ± 0.89	3.31 ± 0.92	ns

## Data Availability

The raw data supporting the conclusions of this article will be made available by the authors on request.

## Supplementary Materials

The following supporting information can be downloaded at: https://www.mdpi.com/article/10.3390/ijms26115099/s1.
