# Supplementary figures and images for: Investigating the Role of Gut Microbiota in the Pathogenesis and Progression of Rheumatoid Arthritis in a Collagen-Induced Arthritis Mouse Model
Source: Int J Mol Sci. 2025 May 26;26(11):5099. doi: 10.3390/ijms26115099 (PMC12154005; doi:10.3390/ijms26115099)

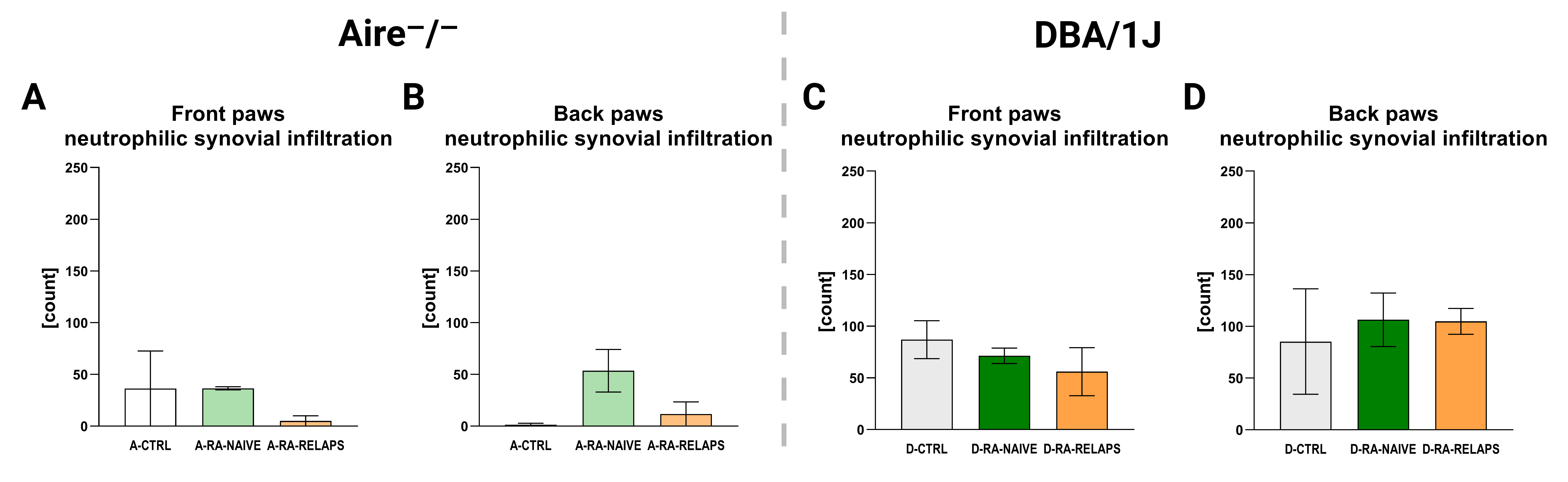

Supplement: Supplementary file 1 [file ijms-26-05099-s001.zip › ijms-3593065-supplementary/FIGURE_S1.png]

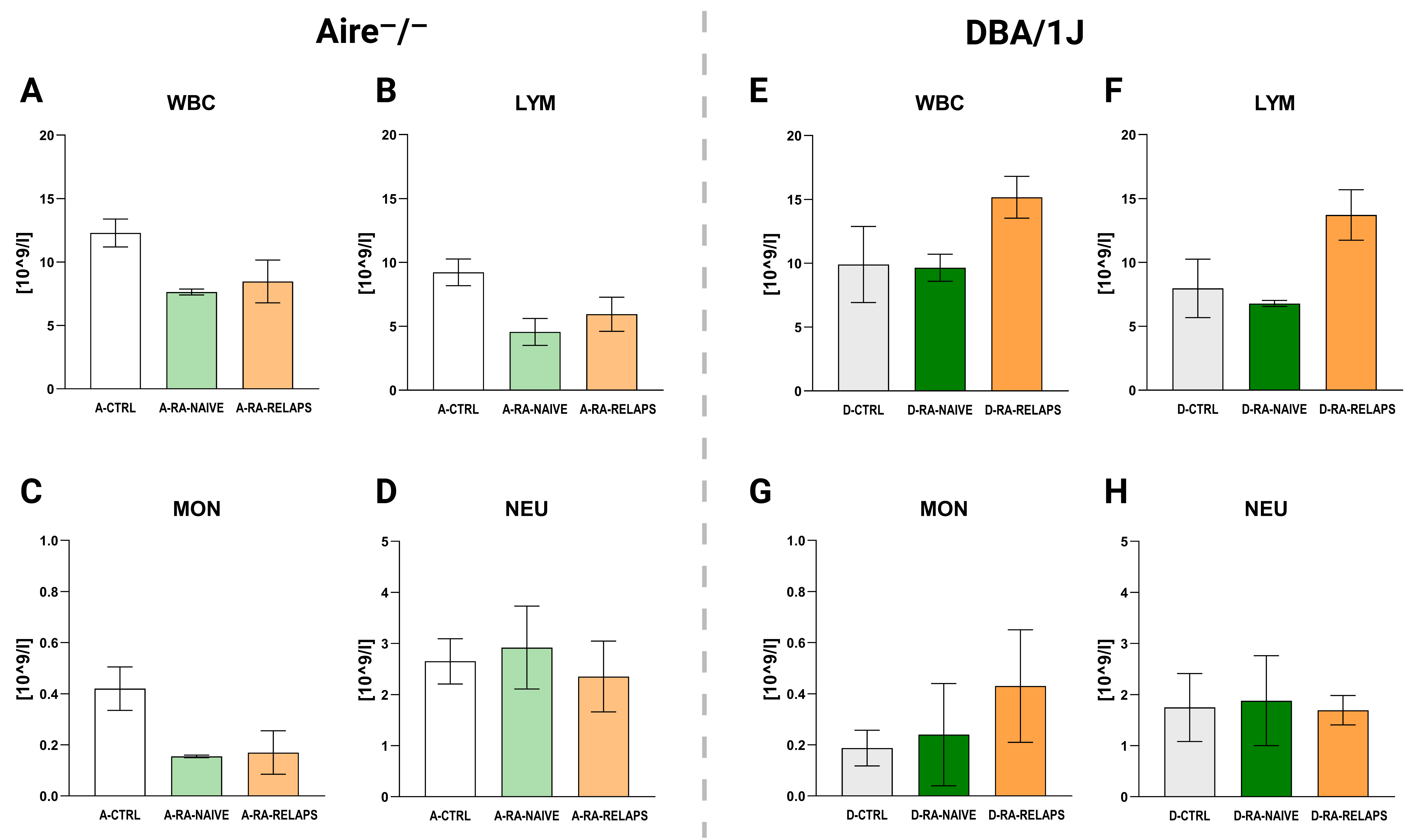

Supplement: Supplementary file 1 [file ijms-26-05099-s001.zip › ijms-3593065-supplementary/FIGURE_S2.png]
